# An Approximate Estimate of the Energy Contained in Strychnine

**Published:** 1893-10

**Authors:** A. D. Barr

**Affiliations:** Calamine, Ark.


### AN APPROXIMATE ESTIMATE OF THE ENERGY CONTAINED IN STRYCHNINE.

By A. D. BARR, M. D.,
CALAMINE, ARK.

In the attempt to estimate the amount of energy manifested by nux vomica and strychnine in the animal organism, the effects produced were carefully noted, and the amount of force necessary to produce such an effect being known, the energy produced was ascertained. It is not claimed that the results set forth in this paper are absolutely correct, but approximately so; and if the effect of all drugs on the system were carefully noted and the force necessary to produce such an effect taken into account, a reliable index and a rational explanation of the action of all drugs would be furnished; and this seems to be the only way to reduce medicine to an exact science. It is self-evident that if the amount of force acting on the animal body to produce a certain diseased condition can be known, and the force a certain drug will liberate when taken into the system, we are placed in a far better position to remedy the evil, or rather to reduce the force to order. As urea is the product of oxidation, and is capable of further oxidation if the amount be decreased, it is evident that the power of oxidation of the system is increased, and as the amount of heat that urea is capable of liberating is known, it is easy to calculate the increased force of oxidation required to produce the decrease.

In my investigation the amount of urea discharged within twenty-four hours was carefully calculated, as it is believed that it furnishes a correct means of knowing the condition of the system. It is scarcely necessary to add that it must be known that the kidneys are in a normal condition, and that the decrease is not mistaken for an accumulation in the system.

By eating the same quality of food and approximately the same amount, the amount of urea discharged during any twenty-four hours will vary but a few grains, and if the amount of food consumed was exact and its chemical composition also the same, the amount of urea would be precise, providing the system remained in the same standard of health. If it be found that the amount of urea discharged for a number of days varies but slightly, then under the administration of a certain drug there is a perceptible decrease, it is a self-evident conclusion that the action of the drug causes the decrease. Strychnine, being composed almost entirely of combustible material, is oxidized when it enters the system, its formula being C 21, H 22, N 2, O 2.

The amount of force a drug is capable of liberating when oxidized in the system is not to be measured by the amount of heat it is capable of liberating when oxidized out of it, for in the system it increases chemical combination so much that a far greater amount of force is manifested than it itself contains.

In seven days when no medicine was taken I found that I excreted 2724.5 grains of urea. In seven in which 170.52 minims of tincture of nux vomica was taken, 2411 grains, showing a reduction of 313.5 grains. In 313.5 grains of urea there are 177.76 heat units Fahr. This converted into force is equal to 68.61031 foot tons Fahr.

Thus 170.52 minims of tincture of nux vomica is capable of liberating the above amount of force when taken into the system and properly assimilated, as is evinced by the increased oxidation of the system, shown by the decreased amount of urea excreted. That nux vomica has the power of decreasing urea is shown by an invariable decrease of that product when the system is under its influence. The decreased amount of urea is almost in definite proportion to the amount of the drug consumed, if sufficient care is taken to both quality and quantity of diet; and if the system did not vary in its power of assimilating, the amount a given quantity of nux vomica would reduce the amount of urea could be exactly ascertained by a mathematical calculation. In my investigation 24.36 minims tincture of nux vomica increased the oxidation of the body 25.53 heat units; and 8.12 minims, the amount taken at once, produced 8.51 heat units Fahr. 25.53 heat units converted into mechanical energy is equal to 9.854 foot tons Fahr., and 8.12 minims to 3.28436 foot tons Fahr.

The amount of heat that 24.36 minims of nux vomica produce would raise the temperature of a body weighing one hundred and fifty pounds 00.227° Fahr., and as this was the increased amount of twenty-four hours, at any given time the increase is 00.009°, an amount imperceptible by the ordinary way of testing temperature As nux vomica is a stimulant the amount of heat it produces is transformed into vital energy as fast as produced, so there is no rise of temperature. The tonic action of nux vomica is explained by its increasing oxidation in a definite degree, in virtue of which the nervous system is enabled to transform heat into nervous energy. To understand how strychnine produces death so quickly it is necessary to consider the amount of force it is capable of liberating when oxidized. One grain of strychnine contains two heat units Fahr.; this converted into the mechanical equivalent of heat is equal to lifting a weight of 154.4 pounds one foot high. One half grain, the smallest fatal dose recorded, is capable, on being oxidized, of liberating a force sufficient to raise seventy-seven pounds one foot high.

When strychnine enters the system it is oxidized, and the force it contains acts on the nitrogenous compounds, and nitrogen being an element of very feeble chemical affinity, it is displaced and allows the carbon, hydrogen and oxygen to rapidly combine, thus producing an enormous amount of force extra to that contained in the strychnine. This force, acting on the nervous system, is so great that it changes its structure so much that it is unable to carry on the vital forces.

That strychnine is oxidized, and causes a rise of temperature can be demonstrated by administering it to animals experimentally. I gave a cat one-half a grain of strychnine; the temperature at the beginning of observation was 100$\frac{4}{5}$° Fahr. The cat was unconscious in ten minutes after taking and in twelve was dead. Temperature at death showed a rise of one-fifth of a degree. The cat weighed three pounds and contained 153.6 heat units. The increase of the temperature of one-fifth of a degree in a body weighing three pounds is equal to .4466 heat units, this converted into mechanical energy is equal to 339.68 foot pounds Fahr., an amount of force sufficient to crush the cat. This was generated, too, by almost instantaneous combustion before the nervous system had time to accommodate itself to the changed condition, and death took place from the system being overwhelmed by the immense force acting on the nervous centers.

